# Cold-induced stress responses during a self-rescue exercise from accidental immersion in ice water in military personnel

**DOI:** 10.3389/fphys.2025.1679550

**Published:** 2025-11-21

**Authors:** Yannick Beres, Raimund Lechner, Elias August, Andreas Koch, Peter Radermacher, Martin Kulla, Enrico Staps

**Affiliations:** 1 Department of Anesthesiology, Intensive Care Medicine, Emergency Medicine and Pain Therapy, Bundeswehr Hospital Ulm, Ulm, Germany; 2 Medical Centre Mittenwald, Medical Service of the German Armed Forces, München, Germany; 3 Section Martime Medicine, German Naval Medical Institute, Kiel-Kronshagen, Germany; 4 Institute of Pathophysiology and Process Development in Anesthesia, University Hospital, Ulm, Germany; 5 Department of Intensive Care and Hyperbaric Medicine, University Hospital, Angers, France; 6 Mountain Infantry Brigade 23, Medical Service of the German Armed Forces, Bad Reichenhall, Germany

**Keywords:** cold stress, cold-water immersion, cold shock response, autonomic conflict, diving reflex, trigeminocardiac reflex, hypothermia, tympanic temperature

## Abstract

**Introduction:**

Cold-water immersion induces autonomic stress responses including sympathetic cold shock and parasympathetic diving and trigeminovagal reflexes, potentially leading to arrhythmias or bronchospasm. Another important complication of cold-water immersion is hypothermia. This study evaluates physiological responses during ice-self-rescue training to assess safety and temperature monitoring accuracy.

**Methods:**

We conducted a prospective observational cohort study of 80 healthy Mountain Infantry soldiers during a standardized ice-self-rescue training in Norway (air temperature −10 °C). Participants underwent partial immersion (0.5 °C water) resulting in transitory submersion (<5 s). They were equipped with continuous 3-lead ECG (n = 34) spirometry (n = 26); and core temperature monitoring (ingestible telemetric capsule (n = 23) versus bilateral tympanic thermometry with and without ear channel occlusion (n = 34). Primary outcomes included cardiac rhythm changes, lung function parameters and temperature measurement agreement.

**Results:**

ECG analysis revealed significant post-immersion tachycardia (median increase of 17 bpm, p = 0.03) and increased RR-interval variability (+90 ms, p < 0.01), without malignant arrhythmias. Spirometry showed no clinically significant changes in FVC or FEV_1_ and peak expiratory flow. Tympanic readings underestimated core temperature post-immersion (median difference −2.8 °C versus capsules, p < 0.01), with ear canal occlusion did not improve accuracy (p = 0.15).

**Conclusion:**

Supervised cold-water immersion during military training exercise elicited expected autonomic stress responses without life-threatening complications in healthy soldiers. These findings suggest structured cold-water training can be safely conducted for fit individuals. Tympanic thermometry proved unreliable following immersion, even after ear channel occlusion. Ingestible capsule thermometry may be a viable approach if invasive measurement is not possible.

**Clinical Trial Registration:**

This trial was registered under “Cold induced stress reactions during cold water immersion” in the German Clinical Trial Register (DRKS) under the registration number DRKS00032345 (https://www.drks.de/search/de/trial/DRKS00032345/details).

## Introduction

Physiologically, cold-water immersion (CWI) elicits a range of autonomic reactions (Berk et al., 1987; Datta and Tipton, 2006; Edmonds et al., 2015; Lott et al., 2021). The initial response to sudden immersion is an involuntary respiratory reaction characterized by rapid, deep inhalation or “gasping”. This initial cold shock response can induce psychologically driven hyperventilation, potentially resulting in numbness, muscle weakness, or loss of consciousness. Hyperventilation-induced hypocapnia leads to respiratory alkalosis, which, in severe cases, may cause cerebral vasoconstriction and unconsciousness. Furthermore, via the Bohr effect, this alkalosis reduces oxygen release to peripheral tissues (Bohr et al., 1904). These acid-base disturbances may also predispose individuals to cardiac arrhythmias. In some cases, bronchospasms may occur (Regnard, 1992). Additionally, declining skin temperature triggers peripheral vasoconstriction, increasing cardiac afterload and myocardial workload, thereby elevating oxygen demand (Giesbrecht, 2016; Kampouri and Vaucher, 2018).

Parasympathetically mediated reflexes such as the diving reflex and the trigeminovagal reflex are activated by cold-water contact with the facial skin, for example, during submersion, leading to a cascade of respiratory, cardiovascular, and vasomotor responses (Kawakami et al., 1967; Gagnon et al., 2013; Lundell et al., 2020). Prolonged immersion further induces hypothermia, which may alter consciousness and provoke electrocardiographic abnormalities, including J waves and ST-segment deviations, ultimately progressing to life-threatening arrhythmias (Kampouri and Vaucher, 2018; Dietrichs et al., 2020).

CWI also precipitates autonomic conflict, characterized by concurrent sympathetic activation (e.g., cold shock, tachycardia) and parasympathetic dominance (e.g., diving reflex-induced bradycardia). This discordance increases the risk of cardiac arrhythmias, such as atrial or ventricular fibrillation, particularly in individuals with preexisting cardiac pathologies. Sudden cold-water exposure represents a high-risk scenario for such events (Lott et al., 2021).

### Objectives

The study’s primary objective was to quantify the physiological and pathophysiological responses of military personnel to falling into ice water and consecutive self-rescue, with particular attention to the potential for adverse effects, including the onset of cardiac arrhythmias. The secondary objective involved evaluating the accuracy and clinical relevance of various temperature measurement methodologies following cold-water exposure.

## Methods

This study was conducted as a prospective observational single cohort study without intervention (Röhrig et al., 2009). It was conducted during the German Armed Forces Mountain Infantry Brigade’s *“*Self-Rescue in an Ice Breakthrough Emergency” training program in Norway with a total of 80 training participants. Since this was a training exercise and the measurements in our feasibility study were only supplementary, it was not possible to perform all measurements on all test subjects. Consequently, ECG data were available in 36 participants (34 included) spirometry in 26 (26 included), ear thermometry in 37 (34 included), and of those, 25 also had continuous core temperature monitoring using an ingestible capsule (23 included).

The training was conducted independently of this study and involved immersion in ice water (0.5 °C) followed by self-rescue and rewarming techniques. A 5 m × 5 m opening was cut into a frozen lake to simulate ice collapse. For the initial ice-self-rescue session analyzed in this study, personnel wore standard field uniforms without insulation and waterproof layer and used ski poles, while skis and backpacks were removed to facilitate safe training. Ambient air temperature was −10 °C. The training protocol followed standardized military guidelines.

Participants were recruited voluntarily from trainees. All volunteers were deemed eligible, as authorization for training included general fitness and cardiac health checkup. All provided informed consent via a standardized document, reviewed and approved by the ethics committee (Reference No. 447/22, University of Ulm) and data protection officer (Bundeswehr Hospital Ulm). Twelve participants had prior cold water immersion training and were analyzes as a subgroup. The research team comprised board-certified military physicians and emergency medicine specialists as well as specialists for anesthesia and for intensive care medicine. All study procedures were conducted in a heated tent adjacent to the training site, maintained at 17 °C. Participants were exposed to ambient temperatures for the majority of the protocol, using the heated tent only for brief data collection. Data collection via ECG and temperature capsules was maintained during the subsequent outdoor period. Ambient conditions included clear skies, no precipitation, and an outside temperature of −10 °C. Participants were assigned to measurement stations consecutively, according to the military training protocol, eliminating researcher bias in the assignment order.

Continuous 3-lead electrocardiogram (ECG) (Spacelabs Healthcare Lifecard CF CE0123, Hawthorne, United States) was performed. ECG analysis focused on 30-s segments pre-, during and post-immersion to assess heart rate (HR) and RR intervals, including the standard deviation of Normal-to-Normal RR intervals (SDNN). Subsequent recordings during the self-rescue phase (initiated at 30 s post-immersion) were excluded due to motion artifact interference. Standard spirometry was performed using a calibrated Vitalograph Pneumotrac-USB spirometer (CE2797, Vitalograph Ltd., Buckingham, United Kingdom). Three consecutive measurements were performed pre- and post-immersion, with the best trial selected to avoid comprehension - or ability-related underperformance. Tympanic temperature was measured bilaterally using an infrared ear thermometer (Braun ThermoScan® PRO 6000, CE 0297, Braun GmbH, Germany, accuracy *0.2 °C)* pre- and post immersion. To evaluate cold-water effects on tympanic readings, the left external auditory canal was occluded with a standard military issued earplug (Honeywell Maximum® Lite Earplugs, Honeywell Automation, Charlotte, United States) while the right remained unsealed. Core temperature was recorded continuously pre-, during and post-immersion via an ingestible telemetric capsule (e-Celsius Performance Capsule, BodyCap, France, accuracy 0.1 °C), using a compatible monitor capable of triplicate parallel measurements. Participants ingested a pre-warmed temperature capsule (equilibrated in a 37 °C water bath) with a sip of tepid water. To ensure stable baseline measurements, oral intake was prohibited thereafter until completion of the following immersion protocol. Blood pressure was not measured during our feasibility study.

Participants performed a simulated ice breakthrough by jumping (fully clothed) in a 5 m × 5 m opening in the lake ice. Immersion time was precisely documented. Transient submersion (<seconds) occurred in most of the participants. Following 30 s of immersion (devoted to breath control, submersion avoidance, and rescue planning), self-rescue was executed using ski poles to climb onto the ice. Post-rescue, participants rolled in snow to remove water from clothing.

The ECG leads were removed immediately after returning to the study tent. Again, tympanic temperatures and three consecutive measurements of spirometry were obtained. Participants then proceeded to the ‘heat preservation’ training module (fire-building and emergency shelter construction).

### Statistical analysis

Statistical analyses were performed using Microsoft Excel (Microsoft Corp, Redmond, WA) and SPSS (Version 30; IBM Corp, Armonk, NY), with normality assessed via Shapiro-Wilk test (α = 0.05). Normally distributed data were compared using Welch’s t-test and presented as Mean and Standard Deviation; non-normal data via Mann-Whitney U tests with exact p-values as Median and Interquartile Range (IQR). Given the sample size, we applied Bessel’s correction (n-1) for variance estimation and reported robust median values with corresponding Interquartile Range (IQR) (Du Prel et al., 2010).

### Data management and storage protocol

All study data were collected and processed according to stringent data protection protocols. The raw datasets, comprising original 3-lead ECG recordings (“Digital Imaging and Communications in Medicine” format), bilateral tympanic temperature measurements and core body temperature telemetry, as well as spirometry data were pseudonymized immediately after acquisition. The pseudonymized datasets were stored on the Bundeswehr’s secure Nextcloud platform. For long-term preservation and analysis, data were archived in their native formats.

We used the STROBE cohort checklist when writing our report (Von Elm et al., 2008).

## Results

### Demographics

Out of 80 participants, ECG data were available in 36, spirometry in 26, and ear thermometry in 37. Within the latter group, 25 also had continuous core temperature monitoring using an ingestible capsule. Two ECG datasets were excluded due to recording artifacts, as well as three ear thermometry and two ingestible capsule datasets because of technical failure. In total, 122 valid measurements were obtained across the different modalities. The mean age of participants was 25.7 years, and 78 (97.5%) of the study subjects were male.

### ECG findings

**FIGURE 1:**
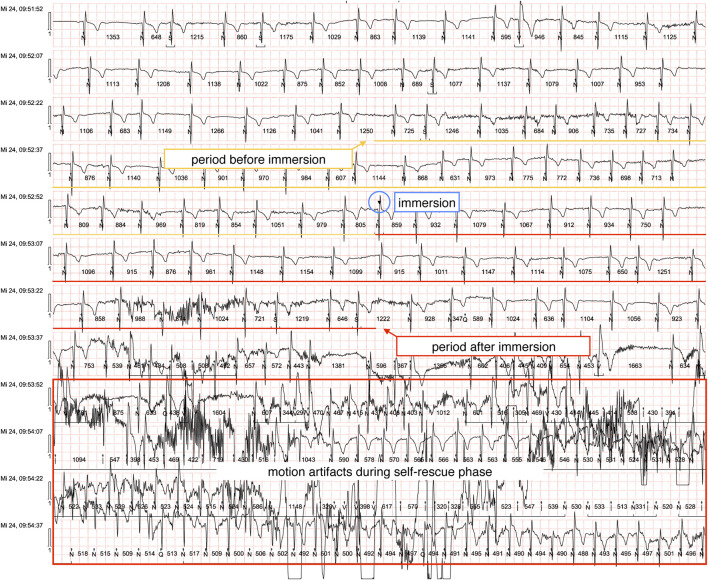
Example ECG with marked evaluation periods (underlined in black).

ECG recordings were successfully obtained from 36 participants, with 34 meeting inclusion criteria following the exclusion of two recordings due to significant motion artifacts. (example ECG shown in Figure 1). All baseline ECGs demonstrated normal sinus rhythm prior to CWI. During the immersion phase, our analysis revealed isolated ventricular extrasystoles (n = 5) occurring in two participants, with no subsequent alterations in QRS morphology or rhythm stability. Notably, we observed no instances of malignant cardiac arrhythmias, including ventricular tachycardia, ventricular fibrillation, or torsade de pointes tachycardias.

Quantitative analysis demonstrated a statistically significant increase in heart rate following immersion (median pre-immersion HR: 114 bpm [IQR 102–129] versus post-immersion HR: 131 bpm [IQR 118–139]; p = 0.0304, paired t-test). Comparative analysis between subgroups revealed no significant difference in heart rate response between participants with prior cold-water immersion experience (n = 12; median HR increase: +14 bpm, [IQR -1 to +17] and first-time participants (n = 22; median HR increase: +17 bpm, [IQR -9 to +27] ]; p = 0.225, Welch’s t-test).

**FIGURE 2:**
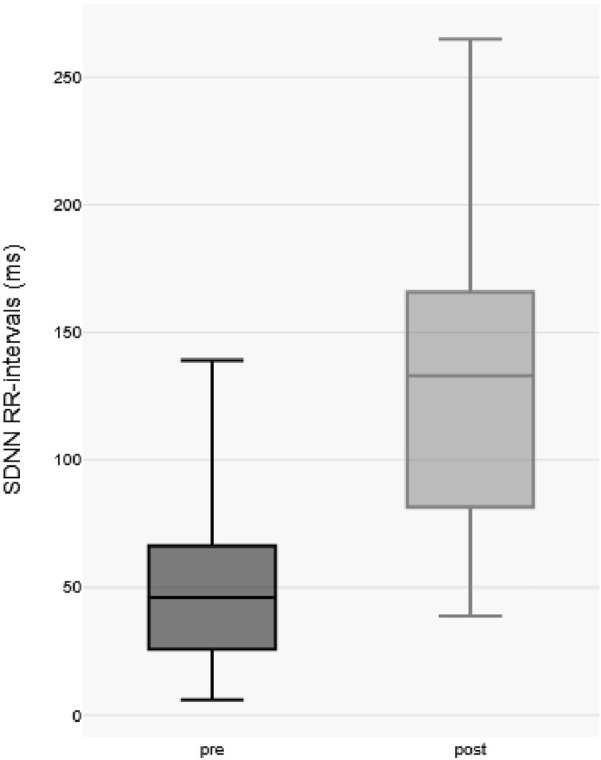
Standard deviation of RR intervals before and after cold water immersion.

Comparative analysis of the standard deviation of Normal-to-Normal RR intervals (SDNN), a well-established metric of heart rate variability, revealed a significant increase in SDNN post-immersion (pre-immersion median: 46.5 ms [IQR 27–71 ms] vs. post-immersion median: 136.5 ms [IQR 83–168 ms]; p < 0.0001 by paired two-tailed t-test), indicating substantial augmentation of parasympathetic nervous system activity following cold exposure (as shown in Figure 2).

There was no evidence of malignant cardiac arrhythmias. Throughout the study, all participants remained hemodynamically stable and free of clinical symptoms.

### Spirometry findings

Lung function parameters were assessed in 26 subjects before and after CWI. Each measurement was analyzed both qualitatively and quantitatively.

As seen in Table 1, no significant differences were observed in forced vital capacity (FVC), forced expiratory volume in one second (FEV_1_), Tiffeneau Index (FEV_1_ Ratio) or mid-expiratory flow (FEF_25_–_75_). A reduction in flow rate was noted in the peripheral airway resistance range (FEF_75_–_85_) and an increase of peak expiratory flow (PEF). None of the participants reported adverse physical symptoms during the observation period.

**TABLE 1:** Results of lung function before and after cold water immersion, mean parameter values with (standard deviation) are shown (number of subjects (n) = 23). FVC: Forced Vital Cpacitiy. FEV1: Expiratory Volume in one second. PEF: peak Expiratory Flow. FEF: Forced Expiratory Flow. IQR: Interquartile range. The data is presented as “median (IQR = interquartile range)” after demonstrating a non-normal distribution with Shapiro-Wilk. P-values were calculated using Mann-Whitney-U-Test.

Measurement	Statistical means	Pre-immersion (n = 23)	Post-immersion (n = 23)	P-values
FVC (L)	Median (IQR)	5.8 (5.4–6.3)	5.8 (5.1–6.3)	0.48
FEV_1_ (L)	Median (IQR)	4.8 (3.9–5.10)	4.6 (4.1–5.0)	0.31
FEV_1_ Ratio	Median (IQR)	0.8 (0.7–0.8)	0.8 (0.7–0.8)	0.47
PEF (L/min)	Median (IQR)	486 (408–531)	417 (363–541)	0.46
FEF25-75 (L/s)	Median (IQR)	4.5 (3.9–5.4)	4.4 (4.0–4.7)	0.18
FEF75-85 (L/s)	Median (IQR)	1.8 (1.5–2.3)	2.21 (1.7–2.5)	0.16

### Temperature findings

Of 25 administered temperature capsules, 23 produced analyzable data, with two exclusions due to telemetry failure. Core temperature baseline was defined as the first stable reading maintained for ≥1 min. The median latency between capsule ingestion and immersion onset was 5:30 min (min: 01:30; max: 13:56).

We obtained complete bilateral tympanic measurements (n = 34 pairs) from 37 attempted recordings, excluding three incomplete datasets. Readings below the Braun ThermoScan® PRO 6000s calibrated range (34 °C–42.2 °C), registering as “LO”; were conservatively recorded as 33.9 °C for analytical purposes. Baseline and post immersion temperatures are summarized in Table 2.

**TABLE 2:** Temperature measurement using temperature capsule (number of subjects (n) = 23) and tympanic measurement (n = 34), in degrees Celcius (°C). The data is presented as “median (IQR = interquartile range)” after demonstrating a non-normal distribution with Shapiro-Wilk. P-values were calculated using Mann-Whitney-U-Test.

Measurement	Statistical means	Pre-immersion	Post-immersion	Temperature difference (Δ)	P-values
Temperature Capsule (°C) (n = 23)	Median (IQR)	37.6 (37.3–37.8)	37.4 (37.2–37.6)	−0.2	0.39
Tympanic Free (°C) (n = 34)	Median (IQR)	36.1 (35.5–36.5)	34.5 (33.9–35.9)	−1.6	0.80
Tympanic Occluded (°C) (n = 34)	Median (IQR)	36.1 (35.5–36.5)	34.7 (33.9–35.1)	−1.4	0.18

Our comparative analysis revealed a statistically significant disparity between core temperature measurements obtained via ingestible capsule versus tympanic thermometry prior to CWI (median Δ +1.5 °C capsule vs. tympanic; p < 0.01, paired two-tailed t-test). Post-immersion, this differential increased substantially (median Δ +2.8 °C), with the capsule-tympanic divergence showing significant augmentation compared to pre-immersion values (p < 0.01, Mann-Whitney U test).

Tympanic measurements demonstrated a significant disparity between pre- and post-immersion measurements (median Δ −1.5 °C p = <0.01) but no significant lateralization between the occluded (earplug) versus non-occluded ear (median interaural difference Δ +0.2 °C; p = 0.15).

**FIGURE 3:**
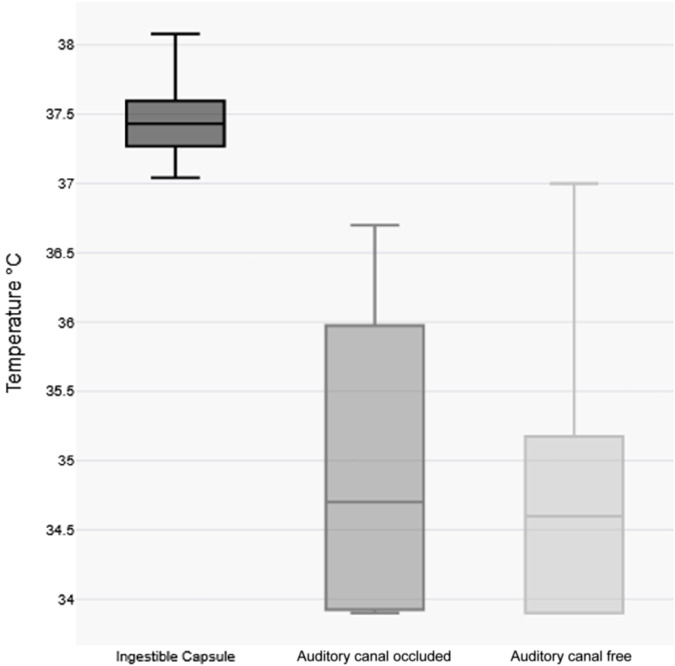
Visualization of variability of temperature measurements post-immersion body temperature Boxplots. Each box represents the interquartile range (IQR) with the median indicated by a horizontal line.

Notably, post-immersion tympanic readings exhibited greater median variability compared to capsule-derived core temperatures as shown in Figure 3.

## Discussion

This pilot study, conducted within the German Armed Forces, aimed to quantify the physiological and pathophysiological responses of military personnel to falling into ice-cold water and performing subsequent self-rescue. The primary objective was to assess the reliability and accuracy of core temperature measurements compared to standard tympanic temperature readings after accidental hypothermia. Additionally, the study focused on evaluating the potential for adverse effects, particularly the onset of cardiac arrhythmias, and other physiological responses under these extreme conditions. Similar studies have been conducted in military personnel (e.g., (Jones et al., 2020; Kelly et al., 2022), However, to the best of our knowledge, this is the first study in a military cohort to directly compare the accuracy of core temperature measurements with standard tympanic temperature readings following accidental hypothermia induced by CWI, as well as to assess the potential risk of arrhythmias during military CWI training.

### ECG changes

Immersion in cold water elicits a robust sympathetic stress response characterized by increased heart rate and elevated blood pressure (Berk et al., 1987; Datta and Tipton, 2006; Edmonds et al., 2015; Giesbrecht, 2016; Lott et al., 2021). Concurrently, parasympathetically mediated cold responses are triggered through the diving reflex and trigeminovagal reflex upon facial skin contact with cold water (Caspers et al., 2011; Giesbrecht, 2016; Buchholz et al., 2017; Dow et al., 2019; Lundell et al., 2020). Our findings demonstrate this sympathetic activation through significant heart rate elevation (median pre-immersion HR: 114 bpm [IQR 102–129] versus post-immersion HR: 131 bpm [IQR 118–139]; p = 0.0304, paired t-test) during immersion, accompanied by increased standard deviation of RR intervals (pre-immersion median: 46.5 ms [IQR 27–71 ms] vs. post-immersion median: 136.5 ms [IQR 83–168 ms]; p < 0.0001 by paired two-tailed t-test), indicating substantial heart rate variability due to parasympathetic activation. This pattern suggests competing autonomic influences: tachypnea from the sympathetic cold shock response may enhance respiratory sinus arrhythmia (Yasuma and Hayano, 2004), while the diving reflex potentially augments vagal tone. Notably, while Finnish naval diver studies report increased parasympathetic activity during prolonged cold exposure (Lundell et al., 2020), we observed no significant bradycardia - likely due to brief submersion durations and the counteracting sympathetic response (Bierens et al., 2016). Known attenuators of the diving reflex, including apnea duration, cognitive distraction, and physical activity (Caspers et al., 2011; Buchholz et al., 2017) may have further contributed to this finding.

The absence of bradycardia underscores the complex autonomic interplay during cold exposure, where simultaneous sympathetic and parasympathetic activation can create autonomic conflict (Shattock and Tipton, 2012; Tipton and Ducharme, 2013; Bierens et al., 2016). Such extreme autonomic responses pose particular risks for individuals with cardiovascular disease (Tso et al., 2022) and may, in rare cases, precipitate life-threatening arrhythmias even in healthy persons (Shattock and Tipton, 2012; Tipton and Ducharme, 2013; Bierens et al., 2016; Lott et al., 2021). Our subgroup analysis of previously CWI trained participants (n = 12) showed no differences in heart rate responses (Δ +14 bpm) compared to novices (n = 22, Δ +17 bpm), though larger controlled studies are needed to establish potential adaptive effects. Current evidence suggests regular cold exposure may enhance parasympathetic responsiveness, though individual variation remains poorly characterized (Jdidi et al., 2024).

Notably, this healthy military cohort without pre-existing cardiac conditions exhibited no malignant arrhythmias, profound bradycardia, or electrocardiographic markers of hypothermia (e.g., Osborn waves) (Tso et al., 2022), demonstrating that supervised CWI can be safely tolerated by fit individuals despite possible autonomic stress.

### Pulmonary function

Exposure to a strong cold stimulus via immersion in cold water initially triggers gasping and an increased respiratory drive. However, if cold exposure persists and core body temperature drops, respiratory drive progressively diminishes (Tipton, 1989; Tipton et al., 1991; Datta and Tipton, 2006; Gagnon et al., 2013; Giesbrecht, 2016).

Cold exposure can induce airway reactions such as bronchoconstriction, bronchial hyperresponsiveness, and even asthma attacks (Carlsen et al., 2008; Näyhä et al., 2011; Muller and Rochoy, 2018). In our cohort, no significant changes were observed in VC, FEV_1_, PEF or mean airway fraction FEF_25_–_75%_. The attenuated reduction as seen in Table 1, contrary to physiological expectations, may be attributable to the physical exertion of participants and subsequent sympathetic activation, who climbed out of the ice hole, rolled in the snow, and returned to the tent. Supporting this, a 2021 Swedish study of healthy subjects demonstrated that short, moderate physical exertion in subzero temperatures does not significantly impair lung function (Eklund et al., 2021). Subgroup analysis of participants who had previously completed *Self-Rescue in an Ice Breakthrough Emergency* training also revealed no significant differences.

### Core body temperature changes

The severity of accidental hypothermia following CWI critically influences treatment algorithms and transport decisions, e.g., ECMO-capabilities Danzl et al., 2016; Paal et al., 2022; Pasquier et al., 2023). The International Commission on Alpine Emergency Medicine (ICAR) recommends esophageal temperature measurement as the reference standard, with the Revised Swiss System serving as an alternative clinical assessment tool when invasive monitoring is unavailable or not suitable in alert patients (Musi et al., 2021; Pasquier et al., 2023).

Although infrared tympanic thermometry represents a rapid, non-invasive technique for temperature assessment, its reliability is significantly compromised in extreme environmental conditions (Danzl et al., 2016; Paal et al., 2022). Accurate measurements require a patent ear canal that is both dry and isolated from ambient conditions (Muth et al., 2010; Musi et al., 2021; Paal et al., 2022). Importantly, most prehospital devices are not validated for use in extreme temperature ranges, further limiting their applicability in CWI scenarios (Henriksson et al., 2017). Despite those findings inadequate thermometers continue to be widely used in extreme environmental conditions, leading to unreliable temperature measurements (Podsiadło et al., 2019; Paal et al., 2022). Temperature capsule measurement in our study design deviated from the typical protocol of ingesting telemetric temperature capsules 2–6 h prior to measurement (Bongers et al., 2015) due to operational constraints. To ensure data reliability temperature capsules were prewarmed in tepid water and we confirmed that the capsules were transmitting stable and physiologically plausible readings before initiating formal data collection. While the two–6-h premeasurement period is standard in research, it may not be practical in hypothermia emergency situations.

Our results demonstrate significant discrepancies between core temperature measurements obtained via ingestible capsule and tympanic thermometry following immersion. The median temperature change was minimal for capsule measurements (Δ −0.2 °C) compared to substantial decreases in both unoccluded (Δ −1.6 °C) and occluded (Δ −1.4 °C) tympanic measurements (p < 0.01). Notably, occlusion of the ear canal with protective plugs failed to improve measurement accuracy, suggesting that even brief head submersion adversely affects tympanic readings. This shows that even in field studies, tympanic temperature measurement is not a suitable means of measuring temperature despite the ear canal being closed with ear plugs the way we did. However, other closure options could in principle sufficiently shield the ear canal from the influence of cold water. This observation aligns with material properties documented in the manufacturer’s safety data: the polyurethane foam used in these earplugs demonstrates hydrophobic characteristics but lacks waterproofing capabilities. While controlled immersion protocols may prevent head submersion, this is rarely achievable in real-world cold-water emergencies.

Several factors likely contribute to the inaccuracy of tympanic measurements under these conditions, including direct water contact during submersion, exposure to cold ambient air and inherent device limitations in extreme environments (Strapazzon et al., 2015; Paal et al., 2022; Pasquier et al., 2023). These measurement errors have important clinical implications: while tympanic readings (median 34.6 °C) would have classified most participants as hypothermic (<35 °C), capsule measurements confirmed normothermic core temperatures (median 37.4 °C). The measured tympanic values may slightly underestimate the true effect of incorrectly low tympanic temperature measurement, as some readings were recorded at the detection threshold (e.g., 33.9 °C). Such discrepancies could lead to inappropriate clinical interventions if treatment decisions were based solely on tympanic assessment.

Based on these findings, we conclude that infrared tympanic thermometry—even with ear canal occlusion—provides unreliable data in CWI scenarios and may lead to erroneous clinical decisions. Besides that, we could show that a short immersion in very cold water (<1 min) with immediate self-rescue has no significant short-term effect on core body temperature. Throughout the observation period no participant met diagnostic criteria for clinical hypothermia as defined by either The Revised Swiss System (Dow et al., 2019) or Wilderness Medical Society guidelines (Musi et al., 2021).

## Conclusion

While cold-water immersion elicits potentially hazardous physiological responses - including the sympathetic cold shock response, parasympathetic diving reflex, and trigeminovagal reflex that may create autonomic conflict - our findings demonstrate that structured training can be safely conducted in healthy volunteers. Notably, we observed no malignant arrhythmias, negative pulmonary changes or life-threatening complications despite the pronounced cardiovascular stress induced by immersion. Furthermore, our results demonstrate that tympanic temperature measurements require careful interpretation in cold environments due to significant measurement variability and potential confounding factors such as ambient temperature and ear canal moisture. Core temperature measurement remains the gold standard in cold environments but can be difficult to obtain in alert patients. The demonstrated limitations highlight the need for further development of robust, non-invasive temperature monitoring solutions suitable for extreme environmental conditions.

## Limitations

This study has inherent limitations: (1) only a small cohort was involved in our feasibility study due to operational constraints in the field, which limits the statistical power of the findings. The prioritization of the training also caused incomplete datasets. While this may affect the generalizability of the results, the data remain valuable for preliminary insights or contextual understanding, provide valuable insight into physiological responses during ice water immersion and demonstrate the feasibility of obtaining such measurements in a realistic training environment; (2) selection bias from using only healthy military personnel limits generalizability; (3) field conditions caused environmental variability; (4) motion artifacts affected ECG data during rescue; and (5) There was no control group in this pilot observational study, as the primary objective of this investigation was to test the measuring devices under training conditions. These limitations were mitigated through standardized protocols and pre/post-immersion analysis.

## Interpretation

Proper planned immersion training of healthy volunteers can safely be performed. A critical clinical implication of our work is the unreliability of tympanic temperature measurements in cold environments. Despite attempts to control for external influences (e.g. ear canal occlusion), tympanic readings significantly underestimated core temperature, potentially leading to misclassification of hypothermia. This reinforces the need for gold-standard core temperature assessment or clinical staging in CWI scenarios, though practical challenges remain in prehospital settings.

## Generalizability

While our findings may not be fully generalizable to all populations or uncontrolled environments, they provide evidence supporting the safety of supervised CWI training in healthy individuals. Future studies should expand upon these results with larger, more diverse cohorts and controlled laboratory conditions to further elucidate the risks and adaptive benefits of cold-water exposure.

## Data Availability

The raw data supporting the conclusions of this article will be made available by the authors, without undue reservation.
